# Relative age effect and performance in elite youth male basketball

**DOI:** 10.1038/s41598-023-31785-4

**Published:** 2023-03-20

**Authors:** Ramazan Tascioglu, Ozan Atalag, Yılmaz Yuksel, Serdar Kocaeksi, Gülsün Güven, Zeki Akyildiz, Hadi Nobari

**Affiliations:** 1 Department of Physical Education and Sports, Graduate School of Health Sciences, Anadolu University, Eskisehir, Turkey; 2 Kinesiology and Exercise Sciences Department, University of Hawai’I at Hilo, Hilo, USA; 3 Galatasaray Football Club, Istanbul, Turkey; 4 Department of Physical Education and Sports Teaching, Faculty of Sport Sciences, Eskişehir Technical University, Eskisehir, Turkey; 5 Department of Coaching Education, Faculty of Sport Sciences, Eskişehir Technical University, Eskisehir, Turkey; 6 Sports Science Department, Gazi University, Ankara, Turkey; 7 Department of Exercise Physiology, Faculty of Educational Sciences and Psychology, University of Mohaghegh Ardabili, Ardabil, 5619911367 Iran; 8 Faculty of Sport Sciences, University of Extremadura, 10003 Cáceres, Spain

**Keywords:** Health care, Diagnosis

## Abstract

The aims of the study were to (i) assess the Relative Age Effect (RAE) on elite young male basketball players, (ii) to analyze whether there is a difference between birth quarters (BQ) according to their minutes played and efficiency ratings as individual performance parameters and, (iii) to analyze the relationship between team efficiency, team success, and RAE with a new approach. The research was conducted on 678 players (Age:15.84 ± 0.42) from 53 teams in six different tournaments (from 2014 to 2019). Although, chi-square test showed that more players were born in first BQ than in all other BQs (p < 0.05), no significant difference between BQs and performance measures was found (p > 0.05). However, a relationship between team RAE score, tournament ranking, and team efficiency score was found (p < 0.05). Furthermore, there was a relationship between tournament rankings and team RAE rankings (p < 0.05). While players born in the first months of the year were more likely to be selected for the national youth teams, performance parameters did not show any significant difference when compared to other players who were born later in the same year. However, results showed that teams that have a higher number of athletes who were born in the earlier months of a year showed higher achievement in the tournaments.

## Introduction

Adolescent athletes are separated into 1 or 2 year age categories in different sports to create a fair competition and placement amongst them. Because of this, athletes with almost one year age difference can be considered as having the same age for selection and/or competition. However, it has been stated that just taking athletes’ chronological age into consideration for team selection might have possible negative consequences in elite sport^1^. Therefore, it was suggested that skeletal age, developmental age, general training age, sport specific training and relative age next to chronological age should be taken into consideration^2^.

Relative age effect (RAE) which effects have been observed in sports^3^, is a fundamental concept within talent identification and it is also a common issue that has been repeatedly addressed in sport science literature. It is a phenomenon known as the difference in athletic performance and developmental stage between players/athletes born in the earlier months of the year and athletes born later during the same year^4^. For instance, in track and field, it was demonstrated that relatively older athletes outperform relatively younger athletes^5^: in male athletes, the estimated performance difference within a given age group decreased consistently with age (i.e., 47.51% at 12 to 5.37% at 17 years old). In other words, there is an asymmetry in the birthdate in favor of relatively older athletes in terms of team building. RAE contributes to the selection of players that are born in the earlier months of the year, and these early-career achievements may contribute to their senior career achievements like winning experiences with the accumulated advantages and snowball effect^2^.

This is especially relevant to the coaches of young teams^2^ who have direct influences on talent identification and team selection process^4^. In this regard, some of the latest developments on this subject have focused on the identifying the role of RAE in team selection process. The occurrence of RAE has been reported on elite young athletes of a number of individual sports such as track & field^6^, judo^7^ and swimming^8^. In addition, RAE has been observed amongst elite young athletes in a range of team sports such as rugby^9^, soccer^10,11^, handball^12,13^, volleyball^14^, and basketball^15–17^.

In basketball, Ibanez et al.^16^ found that RAE was present in all age categories, from under-12 to under-18, with male players born in the first quarter of the year being overrepresented in each category. Similarly, in U16, U18, and U20 European Basketball Championships, Arrieta et al.^15^ found that more male players were born in the first quarter of the year. Both studies showed that while more male players were born in the first quarter, the presence of the relative age effect gradually decreased with increasing age. Moreover, Lupo^18^ studied to determine if the RAE affects the early and late phases of a senior career in elite team sports. They showed that the players born close to the beginning of the year were 1.57 times more likely to reach the first and second Italian divisions of basketball than those born in the last part of the year. In line with this, Riaza et al.^19^ conducted a systematic review that showed no RAE impact on long-term individual performances (success throughout the sport career). In other words, these studies stated that RAE affects the early stages of players' careers, not the late stages, and showed that relatively older basketball players have a better chance of transitioning to their senior careers.

In a meta-analysis by Cobley et al.^3^, gender made little difference to the overall ORs, however basketball as well as ice hockey and soccer were associated with higher occurrences of RAE at both regional and national competitions, and were shown to be vulnerable to RAE for mid to late adolescence (in men aged 15–18 years old). Riaza et al.’s^19^ systematic review supported the RAE's impact on short-term individual performance (in men aged 14–19 years old) and the influences of RAE on basketball competition performance. Therefore, the sample of this research consists of male basketball players between the ages of 15–18. Recent studies on elite young basketball players^15–17^, soccer players^11^, swimmers^8^ and handball players^13^ have demonstrated a more comprehensive understanding of RAE risks by using players' performance statistics.

Despite extensive research on RAE and players' statistics, the literature still remains controversial to show the relationship between team performance and success with RAE^11,13^. In a systematic review of the relationship between performance and RAE, studies were categorized into three groups: studies which show no relationship between the RAE and competition performance; studies which show the influence of RAE reversal (an overrepresentation of athletes who are relatively young or born in the last months of the year) on performance; studies which show an impact of RAE on performance^20^. Therefore, this study had three objectives: (1) To identify RAE in elite young male basketball players, (2) to compare minutes played and player efficiency rating (PER) of players from different BQs as performance parameters, and (3) to investigate the correlation of team PER, team success, and relative age team achievement (RATA) score.

## Methods

### Participants

A total of 678 (age: 15.84 ± 0.42) adolescent male basketball players were included in this study who played in the Turkish Basketball Federation (TBF) International U-16 Men Basketball Tournament for the last six seasons. In the all tournaments, 53 national youth teams from 17 different countries (Argentina, Bosnia and Herzegovina, Bulgaria, China, Croatia, France, Georgia, Germany, Italy, Latvia, Lithuania, Montenegro, Russia, Serbia, Slovenia, Turkey, and Ukraine) competed. All participants were informed of the risks and benefits of participating in the research and provided informed consent before the measurements. The study was performed in accordance with the Declaration of Helsinki and approved by the Local Ethics Committee of the Eskişehir Technical University (No: 87914409-050.99). According to the equation for elite athlete classification which was based on the athletes’ highest standard of performance, their success and experience at that level, the sample group was classified as the semi-elite athletes^21^. Table 1 presents the number of players in the teams and the ranking of teams.

**Table 1:** TBF International U-16 Men Basketball Tournament results (n = 678).

2014–2015 Season*** (n = 146)	2015–2016 Season*** (n = 152)	2016–2017 Season** (n = 104)	2017–2018 Season** (n = 104)	2018–2019 Season* (n = 79)	2019–2020 Season** (n = 93)
Turkey (15)	Lithuania (12)	Turkey U16 (15)	France (12)	Lithuania (12)	Turkey U16 (14)
Lithuania (12)	Serbia (12)	Lithuania (12)	Lithuania (12)	Serbia(13)	Russia (13)
France (12)	Turkey U16 (16)	Argentina (14)	China U17 (11)	Turkey U16 (14)	Lithuania (12)
Italy (12)	Argentina (12)	Latvia (12)	Argentina (12)	France (12)	Slovenia (12)
Bosnia and Herz. (12)	France (12)	Bulgaria (12)	Serbia (12)	Turkey U15 (16)	Serbia (14)
China (11)	Turkey U15 (16)	Georgia (12)	Turkey U16 (16)	Croatia (12)	Latvia (12)
Serbia (12)	Latvia (12)	Ukraine (12)	Bosnia and Herz. (12)		Turkey U15 (16)
Montenegro (12)	Germany (12)	Turkey U15 (15)	Turkey U15 (17)		
Latvia (12)	Montenegro (12)				
Georgia (12)	Ukraine (12)				
Germany (12)	Slovenia (12)				
Russia (12)	Bulgaria (12)				

The number of players in the teams was given in brackets.

*Each team played five games in the season.

**Each team played six games in the season.

***Each team played seven games in the season.

### Data collection

All data was obtained from the official website of Turkish Basketball Federation^22^ at the end of the tournaments. The data included date of birth and game statistics of players. Since the relative age effect was based on months, the date of birth was transformed into birth quarters and age (month) respectively. The BQs were defined as: 1st quarter (BQ1) from January 1st to March 31st, 2nd Quarter (BQ2) from April 1st to June 30rd, 3rd Quarter (BQ3) from July 1st to September 30th, and 4th Quarter (BQ4) from October 1st to December 31st, respectively. The definition is made based on the tournament player participation rule. In other words, the cut-off data of the players' birthdays were checked and they were all on the 1st of January in all countries. Player statistics were used to calculate players’ playing minutes and PER. One thing to note here is that, since playing positions of the players were not available on the official source of the data (Turkish Basketball Federation's website) and we got conflicting clarifications from other sources (coaches and other sources) for playing positions, those were not used in any of the analyses. The study followed the ethical procedures and was approved by the ethics committee of Eskisehir Technical University (No: 87914409-050.99).

### Player efficiency rating

PER formula which was developed by Hollinger^23^ uses positive and negative player statistics and was used as a performance criterion for both players the players^24^ and the teams^25^. Formula aims to calculate a single score of all statistics of the players or teams to be able to evaluate their performance. PER has 12 variables with different coefficients as shown below:

$${\text{Player \, Efficiency \, Rating}}\, = \,{\text{Points}}\, + \,0.{4}\, \times \,{\text{Field \, Goal}}{-\!\!-}0.{7}\, \times \,{\text{Field \, Goal \, Attempt}}{-}\left( {{\text{Free \, Throw \, Attempt}}{-\!\!-}{\text{Free \, Throw}}} \right)\, + \,{\text{Offensive \, Rebound}}\, + \,0.{3}\, \times \,{\text{Defensive \, Rebound}}\, + \,{\text{Steal}}\, + \,0.{7}\, \times \,{\text{Assist}}\, + \,0.{7}\, \times \,{\text{Block}}{-}0.{4}\, \times \,{\text{Foul}}{-}{\text{Turnover}}.$$

### Relative age team achievement (RATA) score

After determining the presence of RAE in terms of players' selection in a team, each player was represented by birth quarter, assuming that the team selected players by their birth quarters. Subsequently, the number of players in each birth quarter was collected and weighed to obtain a RATA score. In addition, the teams were ranked according to their RATA scores to see the relationship between tournament rankings and RATA ranking. This method was used for each team.

Based on the assumption that teams select players by relative age effect quarter, this method has a rationality similar to the Borda Count method used in the voting literature^26^. The difference from the Borda method is that the number 1 is assigned to the last preference instead of 0. The reason for this adaptation in the RATA score is to prevent teams with all players born in the BQ4 from having a total score of 0. An example of the proposed method for RAE on team sports is given below:

**Table:** 

Players (P)	Players’ BQs				
P1	BQ1				
P2	BQ1	BQ1	BQ2	BQ3	BQ4
P3	BQ1	7	4	0	1
P4	BQ1				
P5	BQ2	= 4*BQ1 + 3*BQ2 + 2*BQ3 + 1*BQ4			
P6	BQ4				
P7	BQ1	= 4*7 + 3*4 + 2*0 + 1*1			
P8	BQ1				
P9	BQ2	RATA score = 41			
P10	BQ1				
P11	BQ2				
P12	BQ2				

### Statistical analysis

Chi-square test was used to compare differences between the observed and expected birth quarter distributions for selected players to the teams for each season (25% for BQ1, BQ2, BQ3, and BQ4). The odds ratios (OR) with thresholds 1.22, 1.86, and 3.00 as benchmarks for small, medium, and large effect sizes, respectively and 95% confidence intervals (CI) were used^27^. OR and 95% CI were calculated by comparing quarters to BQ4 (i.e. BQ1 versus BQ4, BQ2 versus BQ4, BQ3 versus BQ4). This is an effective way to show inequalities and the effect size of RAES^3,7^. Performance parameters were players' playing minutes (as seconds) and PER. One-way ANOVA was used to assess differences between players’ minutes played. PER was calculated with the total statistics of each player at the end of the tournament. Since the number of matches was different in each season, PER scores were standardized by dividing it with the number of matches. Covariance analysis (ANCOVA) were used to assess differences between birth quarters' PERs with two covariance [players’ playing time (s) and age (month)]. Data of PER with non-normal distribution was transformed (by using square root technique) before further parametric analysis was done^28^. All data was found normally distributed (Kolmogorov–Smirnov normality test) and homogenous (Levene’s test of homogeneity variances). Cohen’s (1988) effect sizes (ESs) and thresholds (0.00, 0.06 and 0.14 as benchmarks for small, medium, and large effect sizes, respectively) were used to compare the magnitude of the differences in performance parameters (minutes played and PER) between BQs. Partial eta squared value^29^ was used to show how much of the variance in the players’ efficiency rating was explained by the birth quarters. Partial eta squared value was converted to a percentage by multiplying it by 100^28^. Assumptions of normality, homoscedasticity, and linearity were checked for correlation analysis^28^. Kendall's tau b correlation coefficient was used, which gave better results than Spearman's rho in the linked ranks^30^. Coefficient of determination (r^2^) were used to check for variance in variables shared^28^. SPSS 25^®^ software was used for the statistical analysis, and a significance level of p < 0.05 was adopted for all tests.

### Ethics approval and consent to participate

The study was conducted according to the guidelines of the Declaration of Helsinki and approved by the Eskişehir Technical University Review Board (No: 87914409-050.99). Written informed consent was obtained from the participants to publish this paper.

## Results

Chi-square results showed a different distribution from expected in favor of first BQs both in all data (χ^2^ = 153.788; p < 0.001) and all seasons (χ^2^ = 47.92; χ^2^ = 23.53 χ^2^ = 21; χ^2^ = 30.54; χ^2^ = 21; χ^2^ = 24.3, p < 0.001). In other words, more basketball players were born in the first BQs. The ORs results showed significant and large ORs (ranging from 3.07 to 6.27) for players born in Q1 and Q4, significant and medium and large ORs for players born in Q2 and Q4 (ranging from 2.69 to 4.17), and for players born in Q2 and Q4 (ranging from 1.23 to 2.73) (Table 2).

**Table 2:** RAE frequency for each comparison by birth quarters (BQ1, BQ2, BQ3, BQ4), with odds ratio (OR) and 95% confidence intervals (CI).

Seasons	BQ1	BQ2	BQ3	BQ4	Exp	χ^2^	p	BQ1 vs BQ4, OR (CI)	BQ2 vs BQ4, OR (CI)	BQ3 vs BQ4, OR (CI)
All	278	206	132	62	169.5	153.78***	0.00	4.48 (3.28–6.14)	3.32 (2.38–4.62)	2.12 (1.49–3.06)
2014–2015	69	36	30	11	36.5	47.92***	0.00	6.27 (3.06–12.85)	3.27 (1.49–7.19)	2.73 (1.21–6.17)
2015–2016	52	49	37	14	38	23.53***	0.00	3.71 (1.88–7.34)	3.50 (1.76–6.97)	2.64 (1.28–5.47)
2016–2017	40	35	16	13	26	21***	0.00	3.07 (1.48–6.40)	2.69 (1.27–5.71)	1.23 (.50–3.01)
2017–2018	47	28	21	8	26	30.54***	0.00	5.87 (2.54–13.58)	3.50 (1.41–8.71)	2.62 (1.00–6.88)
2018–2019	33	25	15	6	19.8	21***	0.00	5.50 (2.07–14.60)	4.17 (1.50–11.56)	2.50 (0.81–7.69)
2019–2020	37	33	13	10	23.3	24.3***	0.00	3.70 (1.65–8.29)	3.30 (1.45–7.52)	1.30 (0.47–3.56)

***p < 0.001.

No significant difference between birth quarters on players’ minutes played was found for all (p = 0.32), 2014–2015 season (p = 0.26), 2015–2016 season (p = 0.54), 2016–2017 season (p = 0.71), 2017–2018 season (p = 0.95), 2018–2019 season (p = 0.11), 2019–2020 season (p = 0.14) (Table 3).

**Table 3:** One way analysis of variance for minutes played of players by birth quarters.

Seasons	Age year (mean ± SD)	BQ	n	Mean ± SD	95% CI	F	P	η^2^
All	15.84 ± .42	BQ1	278	1049.46 ± 437.60	997.79–1101.13	1.16	0.32	0.00
		BQ2	206	1017.80 ± 425.55	959.34–1076.25			
		BQ3	132	998.06 ± 446.02	921.26–1074.85			
		BQ4	62	944.50 ± 430.57	835.15–1053.05			
2014–2015	15.96 ± .23	BQ1	69	1135.87 ± 480.42	1020.46–1251.28	1.34	0.26	0.02
		BQ2	36	1066.11 ± 423.22	922.91–1209.31			
		BQ3	30	940.80 ± 499.42	754.31–1127.29			
		BQ4	11	986.55 ± 399.78	717.97–1255.12			
2015–2016	15.82 ± .39	BQ1	52	934.46 ± 484.68	799.53–1069.40	0.72	0.54	0.01
		BQ2	49	1056.71 ± 494.67	914.63–1198.80			
		BQ3	37	981.38 ± 427.41	838.87–1123.88			
		BQ4	14	1083.93 ± 503.78	793.06–1374.80			
2016–2017	15.78 ± .44	BQ1	40	1017.15 ± 379.81	895.68–1138.62	0.45	0.71	0.01
		BQ2	35	1055.54 ± 352.80	934.35–1176.73			
		BQ3	16	1088.75 ± 380.94	885.76–1291.74			
		BQ4	13	937.92 ± 430.47	677.79–1198.05			
2017–2018	15.91 ± .52	BQ1	47	1036.66 ± 486.31	893.87–1179.45	0.10	0.95	0.00
		BQ2	28	989.25 ± 386.04	839.56–1138.94			
		BQ3	21	1024.38 ± 489.54	801.55–1247.22			
		BQ4	8	958.50 ± 394.39	628.78–1288.22			
2018–2019	15.75 ± .44	BQ1	33	1056.27 ± 365.63	926.63–1185.92	2.04	0.11	0.07
		BQ2	25	976.12 ± 443.94	792.87–1159.37			
		BQ3	15	925.20 ± 390.32	709.05–1141.35			
		BQ4	6	619.83 ± 494.99	100.38–1139.29			
2019–2020	15.78 ± .47	BQ1	37	1095.08 ± 300.71	994.82–1195.34	1.84	0.14	0.05
		BQ2	33	923.09 ± 416.61	775.37–1070.81			
		BQ3	13	1107.62 ± 470.68	823.19–1392.05			
		BQ4	10	895.20 ± 320.32	666.06–1124.34			

According to the results of the covariance analysis, when the birth quarters were controlled, there was a significant relationship between the players' efficiency rating, minutes played and age. Meaning, players’ efficiency ratings are influenced by the minutes played and the age of the players. In other words, whether there is a significant relationship between the covariate and the dependent variable while controlling for the independent variable was checked during the ANCOVA process. It was found that playing time (sec) and age (month) together explained 50% of the variance in the dependent variable. After adjusting for minutes played and age of players, there was no significant difference between birth quarters (p > 0.20) (Table 4).

**Table 4:** Covariance analysis for efficiency rating by birth quarters.

Source	SS	df	MS	F	p	η2
Age (month)	4.17	1	4.17	11.71	0.00	0.02
Minutes played (s)	193.82	1	193.82	542.34	0.00	0.48
Birth quarters	1.65	3	0.55	1.56	0.20	0.00
Error	206.54	579	0.36			
Total	406.18	584				

Table 5 presents the results of adjusted efficiency ratings by controlling minutes played and the age of the players.

**Table 5:** Means and adjusted means of players’ efficiency ratings.

Birth quarters	n	Mean	Adjusted mean	95% CI
January–March	245	1.90	1.80	1.72–1.89
April–June	171	1.90	1.90	1.81–1.99
July–September	116	1.85	1.95	1.84–2.06
October–December	53	1.70	1.94	1.77–2.11

Table 6 presents the Kendall’s tau b correlation results of RATA score and team efficiency and success relationship. There was a small negative correlation between RATA score and tournament ranking (r = −0.20, p = 0.04, r^2^ = 0.04) and small positive correlation between RATA score and team efficiency score (r = 0.20, p = 0.04, r^2^ = 0.04). They shared 4% variance. There was also a small positive correlation between tournament ranking and RATA ranking (r = 0.26, p = 0.02 r^2^ = 0.07). They shared 7% variance (Table 6).

**Table 6:** RATA score and team efficiency and success relation (n = 53).

Team scores	1	2	3	4
1. RATA score	–			
2. Team ranking	−0.20*	–		
3. RATA ranking	−0.87***	0.26*	–	
4. Team efficiency score	0.20*	−0.60***	−0.23*	–

*p < 0.05, **p < 0.01, ***p < 0.001.

## Discussion

The aim of this study was to examine the relationship between the relative age effect and performance of elite young male basketball players individually and as a team. The results of this study showed that RAE was apparent for selection of young basketball players who participated in these international tournaments. However, players’ individual performances were not found to be correlated with RAE. In other words, players who were born in the earlier quarters of the year had similar values in terms of playing times and player efficiency ratings compared to the players who were born later during the year. However, building a team with athletes born in the first months of the year was found to be related to achieve a higher position in tournaments. In other words, while the RAE did not change the individual performances of basketball players; it changed team performances in a positive way.

Studies involving U16 elite athletes from individual and team sports showed that RAE is significant^6–8,10,12,14,16^. Studies also showed that there are significant physical changes at these ages. Tanner and Whitehouse^31^ stated that in the 16 year-old male population, the difference in height might reach up to 16 inches in a year time. Accordingly, Delorme and Raspaud^32^ found that basketball players who were born in the first and second quarter of the year are taller than players who were born later during a year. These findings all indicate that physical attributes can significantly change in a one-year period.

Although RAE was significant in all studies done on U-16 elite basketball athletes, different findings were found in the relationship between the players' individual performances and the RAE. While one study on elite young basketball players found a relationship between the RAE and the performance index rating (PIR)^16^; other studies have found no relationship between RAE and PIR^15,17^. However, Arrieta et al.^15^, found a relationship between playing time as a performance parameter and RAE. In the present study, there was no relation found between RAE and both playing time and PER.

In addition to the studies on the relationship between players' individual performances and RAE, some studies aimed to reveal the relationship between RAE and team performances^11,13,17^. In these studies investigating the relationship between the rankings of the teams at the end of the season and the RAE, a relationship was found in soccer^11^ and in basketball^17^, but not in handball^13^. In the present study, a relationship was found between when using both the old (team ranking and team efficiency score) and new approaches (RATA ranking and RATA score) in terms of team performance. Considering that the high-level competition experience obtained in the youth championships allows the national teams to compete at the final levels of the senior championships^33^, the correlation shows which national basketball teams may have these achievements both in their youth and senior careers.

A possible limitation of the current research is the evaluation of only national youth teams in the tournament. Delorme, Boiché, and Raspaud^34^ noted that if there is a biased distribution among the entire population of licensed players in a single sport, it may occur at elite levels as well. Regardless, present study sample consisted of 53 teams from 17 different countries in six seasons of the tournaments played and that provided a relatively wide perspective for analysis.

## Practical application/recommendations

With respect to the current results of RAEs, basketball coaches and governing bodies should consider strategies that provide balance on early specialization and competitive team success as seen in the tournaments in recent years. Some suggestions to counteract and eliminate RAEs in the literature include age-group bandwidths, bio-banding, corrective adjustment procedures (CAPs), and current and potential squad approaches^2,3,5^. Turkish Basketball Federation made a decision in the past years to participate in the recent tournaments with two teams with “current” and “potential” squads based on athletes’ current abilities and potential abilities^35^. Considering the difficulties of using age-group bandwidths, bio-banding, and CAPs in terms of team sports, current and potential team approaches can be used in team sports, as TBF did.

Besides the success ranking approach used in the previous studies in the RAE literature for team success, “the assigning weightage” approach was used in this present study, and to our understanding, that was not implemented in the previous studies before. Advanced analysis can be used with this new approach, which allows creating continuous variables from ordinal variables.

## Conclusions

One of the main results of this study that players who were born in the last quarter of the year were secondary during the player selection process. According to Merton^36^ who also coined the term “Matthew effect” (a situation in which a child given early developmental advantages through sport is simultaneously set up for success in other areas), this might also cause young kids who are born in the later months to miss out on social and decision making aspects of life as well (such as dealing with loss after a game).

Another important result of this research is that, although RAE did not affect the individual performances of basketball players, it affected team performance. According to the literature, “special” or “chosen” kids are still likely to have more opportunities for success as accumulated advantage, also known as the “snowball effect”^2^. This result shows that the snowball effect is not only limited to their accumulated advantages for their own future, but also helps them to experience opportunities for success in the moments they are together.

RAE and objective measures of game sense are listed among the features that should be considered in effective talent development. In addition, it was emphasized that talent development problems caused by RAE are not only specific to team building, but they affect talent selection process as well. In most cases, talent selection takes a considerable time of team staff and uses a significant portion of team’s budget. That is why, these fore-mentioned effects should be taken into account in long-term player development and when selecting the young players for both international and national competitions^37^.

## Data Availability

The data presented in this study are available on website: https://osf.io/9rgk8/ with Identifier: https://doi.org/10.17605/OSF.IO/9RGK8.
